# Microfluidic Mobility Shift Assay for Real-Time Analysis of Peptide *N*-Palmitoylation

**DOI:** 10.1177/2472555216689529

**Published:** 2017-01-31

**Authors:** Thomas Lanyon-Hogg, Neki V. Patel, Markus Ritzefeld, Katherine J. Boxall, Rosemary Burke, Julian Blagg, Anthony I. Magee, Edward W. Tate

**Affiliations:** 1 Department of Chemistry, Imperial College London, London, UK; 2 Cancer Research UK Cancer Therapeutics Unit, The Institute of Cancer Research, London, UK; 3 Molecular Medicine Section, National Heart & Lung Institute, Imperial College London, London, UK

**Keywords:** enzyme assays or enzyme kinetics, lipids or lipid metabolism, fluorescence methods, medicinal chemistry

## Abstract

The Hedgehog pathway is a key developmental signaling pathway but is also implicated in many types of cancer. The extracellular signaling protein Sonic hedgehog (Shh) requires dual lipidation for functional signaling, whereby N-terminal palmitoylation is performed by the enzyme Hedgehog acyltransferase (Hhat). Hhat is an attractive target for small-molecule inhibition to arrest Hedgehog signaling, and methods for assaying Hhat activity are central to understanding its function. However, all existing assays to quantify lipidation of peptides suffer limitations, such as safety hazards, high costs, extensive manual handling, restriction to stopped-assay measurements, or indirect assessment of lipidation. To address these limitations, we developed a microfluidic mobility shift assay (MSA) to analyze Shh palmitoylation. MSA allowed separation of fluorescently labeled Shh amine-substrate and palmitoylated Shh amide-product peptides based on differences in charge and hydrodynamic radius, coupled with online fluorescence intensity measurements for quantification. The MSA format was employed to study Hhat-catalyzed reactions, investigate Hhat kinetics, and determine small-molecule inhibitor IC_50_ values. Both real-time and stopped assays were performed, with the latter achieved via addition of excess unlabeled Shh peptide. The MSA format therefore allows direct and real-time fluorescence-based measurement of acylation and represents a powerful alternative technique in the study of *N*-lipidation.

## Introduction

The Hedgehog (Hh) signaling pathway is a key determinant of embryogenesis, and knockout of signaling pathway components results in severe developmental defects or neonatal lethality.^1^ Hh signaling is commonly deactivated in healthy adult tissues, but signaling is also aberrantly reactivated in various malignancies including pancreatic, lung, and breast cancers.^2,3^

Hh signaling is mediated by three homologous secreted proteins (Sonic [Shh], Desert [Dhh], and Indian [Ihh] hedgehog), all of which require dual lipidation for function. Hh proteins exhibit a unique autocatalytic intein-like cleavage step during protein maturation, mediated by a C-terminal processing domain, that results in the formation of a C-terminal cholesteryl ester.^4^ In addition, cleavage of an N-terminal signaling peptide from the precursor protein exposes an N-terminal cysteine residue that is subsequently palmitoylated by the membrane-bound *O*-acyltransferase enzyme Hedgehog acyltransferase (Hhat) in the endoplasmic reticulum.^5^ The purpose of these lipidation events is yet to be fully determined; current evidence indicates lipidation plays a role in the assembly of multimeric extracellular Shh signaling particles and affects the distance over which signaling can occur.^6^ *N*-palmitoylation is critical for signaling, with Hhat knockout mice exhibiting similar phenotypes to Shh knockout mice.^7^

Hhat can be viewed as an attractive target to block Hh signaling through affecting signal-transmitting cells rather than signal-receiving cells.^6^ Indeed, a recently identified small-molecule inhibitor of Hhat, named RUSKI-43, is proposed as a potential therapeutic agent to block Hh signaling in breast and pancreatic cancers.^8,9^ This molecule, however, exhibits unspecified effects outside of the canonical Hh signaling pathway.^9,10^ Indeed, our own data demonstrate that RUSKI-43 inhibits Hhat in biochemical assays,^11^ whereas in cell-based assays, the compound exhibits significant off-target effects and general cytotoxicity, thereby precluding its use as a selective chemical probe for Hhat in cells.^12^ Alternative inhibitors, such as RUSKI-201, display higher potency and an on-target mode of action in cells^12^; however, further investigation is required to determine the pharmacophore of these molecules and to improve their profile as chemical probes for Hhat function. Such investigations necessitate the support of biochemical assays that are robust, facile, and cost-effective.

Previous studies of Hhat have employed ^125^iodine-radiolabeled palmitic acid, with either phosphorimaging or scintillation counting detection.^8,13,14^ Such assays are, however, limited in their utility owing to safety, environmental, and cost issues associated with radioactive substrates. The advent of bio-orthogonal alkyne- or azide-tagged fatty acids in combination with “click chemistry” functionalization has greatly enhanced lipidation studies.^15^ Recently, our group has developed a click chemistry–armed enzyme-linked immunosorbent assay (click-ELISA) to measure palmitoylation of Shh by Hhat.^11,16^ Here, a biotinylated Shh peptide is labeled with an alkyne-tagged palmitate analogue (YnPal); the alkyne group is then functionalized with an azido-FLAG peptide to allow standard ELISA-based detection.^11^ Although this method is not hindered by the caveats of employing radiolabeled substrates, it necessitates multiple liquid-handling steps, which limit throughput.

There is therefore an unmet need for new assay formats to study palmitoylation by Hhat that overcome these existing issues. To this end, we developed a microfluidic mobility shift assay (MSA) that allows fluorescence-based measurement of Shh palmitoylation. This format allows automated, real-time monitoring of the peptide lipidation reaction, determination of kinetic parameters, and dose-response analysis of small-molecule inhibitors, which may prove extendable to other classes of *N*-lipidation.

## Materials and Methods

### Recombinant Protein Expression

HEK293a cells stably expressing Hhat-FLAG-His_8_ were cultured, and dodecylmaltoside (DDM)-solubilized membrane fractions (P100(sol)) were prepared as previously described.^11,13^ Further purification of Hhat from the P100(sol) fraction (8 mL) was performed by dilution to 0.2% DDM (w/v) in solubilization buffer (20 mM HEPES, pH 7.3, 350 mM NaCl, 5% [v/v] glycerol, 42 mL) followed by incubation with nickel-NTA resin (Sigma, St. Louis, MO; 1 mL) for 2 h. The resin was separated and washed with 0.15% DDM solubilization buffer containing 20 mM imidazole (10 mL) followed by 0.1% DDM solubilization buffer containing 20 mM imidazole (10 mL). Bound proteins were eluted with 0.1% DDM solubilization buffer containing 500 mM imidazole (4 × 1 mL). Elution fractions were pooled and subsequently concentrated using an Amicon Ultra spin column (10 kDa molecular weight cutoff; Merck Millipore, Billerica, MA). Buffer exchange was performed using repeated dilution-concentration cycles with DDM-free solubilization buffer to achieve >1000-fold dilution of imidazole. The concentrated Hhat-enriched P100(sol) fractions were diluted to a final volume of 400 µL, flash frozen, and stored at -80 °C. Protein concentrations were estimated using the Bio-Rad DC protein estimation kit (Bio-Rad, Hercules, CA) and adjusted to 0.45 mg/mL. All purification and protein manipulation steps were performed at 4 °C.

### Chemical Synthesis

Palmitoyl-CoA (Sigma) was reconstituted in 20 mM NaOAc (pH 6.0) and stored at -20 °C. Unlabeled and carboxyfluorescein (FAM)–labeled Shh peptides were synthesized using standard solid-phase peptide synthesis protocols. Full details of synthesis and characterization of peptides are provided in the Supplemental Information. For enzyme studies, FAM-labeled peptide concentrations were determined through A_495_ measurement in 0.1 M Tris-HCl (pH 8.0) on a PerkinElmer (Waltham, MA) Nanodrop ND-100 spectrophotometer using a FAM molar extinction coefficient of 83,000 M^−1^cm^−1^.^17^ RUSKI-41, RUSKI-43, RUSKI-101, and RUSKI-201 were synthesized as previously described^18,19^ and stored as 10 mM DMSO stocks at -20 °C.

### Microfluidic MSA

Prior to use, all buffers were passed through a 0.2-µm filter, and all reagents were cleared via centrifugation (5 min, 16,000 rcf, 4 °C). Reaction mixtures were prepared in reaction buffer (100 mM 2-(*N*-morpholino)ethanesulfonic acid, pH 6.5, 20 mM NaCl, 1 mM dithiothreitol [DTT], 1 mM tris(2-carboxyethyl)phosphine [TCEP], 0.1% [w/v] bovine serum albumin) in 384-well low-volume plates (Corning, Corning, NY). Shh(1-10)-FAM (2 µM, 12.5 µL) was incubated with Hhat-enriched P100(sol) (0.01–0.45 mg/mL, 2.5 µL), and the reaction was started by the addition of palmitoyl-CoA (7.5 µM, 10 µL). Assays were analyzed on a PerkinElmer 122919 LabChip EZ Reader using a 12-sipper chip. Reaction mixtures were separated using a −3000 V upstream voltage and −500 V downstream voltage at −1.7 PSI screening pressure. Sample sips of 0.2 s were separated by at least 60 s in kinetic mode. Under stopped conditions, the enzyme reaction was halted by the addition of a 20-fold excess of unlabeled Shh peptide (220 µM, 2.5 µL) in reaction buffer at required time points.

For dose-response analysis, serial dilutions of RUSKI inhibitors were performed using a Labcyte Echo550 liquid handler with DMSO backfill to a total volume of 250 nL. Measurements were background corrected against heat-inactivated Hhat-enriched membrane fraction and normalized to DMSO-only control samples. Raw fluorescence peak area data were processed to percentage conversions using LabChip EZ Reader software and evaluated using GraphPad Prism 6.0. Dose-response nonlinear regression was performed via a three-parameter fit, with the lower plateau constrained to the maximum inhibition value of RUSKI-201.

## Results

### Microfluidic Mobility Shift Resolution of Shh Peptides

**Figure 1.**
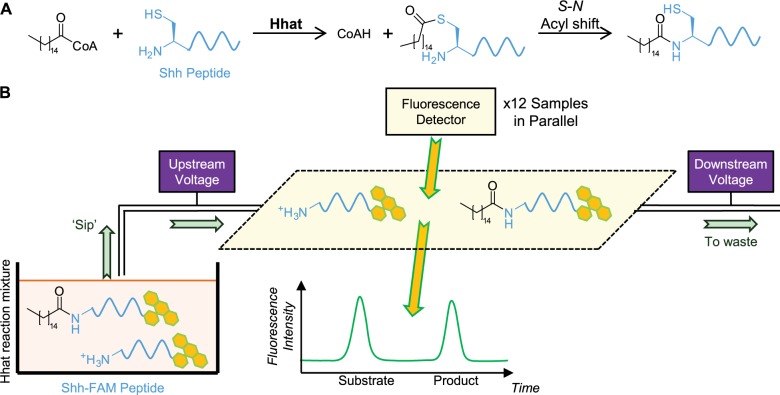
Shh *N*-palmitoylation by Hhat and schematic representation of the microfluidic mobility shift assay (MSA). (**A**) The Shh N-terminal cysteine is palmitoylated on the thiol side chain and subsequently undergoes *S,N*-acyl shift to afford the *N*-acetylated Shh product. (**B**) Shh palmitoylation reactions contain FAM-labeled substrate amine and product amide species. Low-volume (nL) “sips” of the reaction mixture are passed through a microfluidic tube across a potential difference, separating peptides on the basis of charge in aqueous solution. Peptides are quantified on the basis of fluorescent peak area to determine substrate turnover. The MSA format allows analysis of up to 12 reaction mixtures in parallel. CoA = coenzyme A.

Posttranslational palmitoylation is most often observed on cysteine residues^15^; however, *S*-palmitoylation of the N-terminal cysteine residue of Shh is unusual as the thioester is proposed to undergo rearrangement through an *S*-*N* acyl shift to form an amide-linked palmitoyl conjugate (**Fig. 1A**).^13^ We therefore sought to exploit the hydrodynamic radius and charge differences between amine-substrate (RNH_3_^+^) and amide-product (RNHCOR′) Shh peptides in aqueous solution to facilitate separation through a microfluidic MSA. In MSA, the reaction mixture is sampled (“sipped”) into the microtube by application of negative pressure within the tube. Electrophoretic mobility shift is then achieved by application of a voltage potential difference across the microtube to separate molecules of different charge by electrostatic interactions as they pass through (**Fig. 1B**).^20^ Online fluorescence detection enables quantification of the two FAM-labeled peptide species. Recording the fluorescence emission over time and calculation of peak areas determines the percentage substrate conversion.

The MSA format has found particular utility in phosphatase and kinase activity assays, in which addition or removal of the phosphate moiety results in a charge difference of ±2.^21,22^ Software is available to predict screening voltages and pressures for phosphorylation of peptide sequences to obtain separation of substrate and product peaks. In the case of *N*-palmitoylation, no such predictive software is currently available; therefore, three peptide sequences of differing net charges were synthesized as substrate and palmitoylated product species (**Suppl. Table S1**). Product-substrate mixtures were then tested for separation under a range of voltages and pressures (**Suppl. Table S2**; **Suppl. Figs. S1–S3**). These iterations identified that maximal separation was afforded by the human Shh peptide sequence H_2_N-**CGPGRGFGKR**(K-FAM)G-CONH_2_ (Shh(1-10)-FAM) and sampled for 0.2 s at −3000 V and −500 V upstream and downstream voltages, respectively, and at −1.7 PSI screening pressure. This peptide sequence also represented the maximum coverage of the native Shh sequence (residues 1–10) of the peptides tested. The ability of MSA to separate palmitoylated and nonpalmitoylated Shh peptides indicated that this assay could be employed as a new method to measure Hhat activity.

### Development of an Hhat MSA

**Figure 2.**
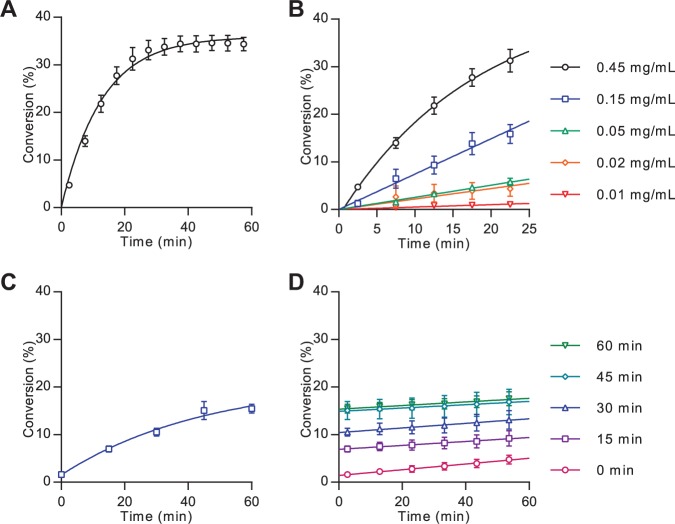
Mobility shift assay analysis of Hhat-mediated Shh palmitoylation. Analysis of Hhat-catalyzed lipidation was performed as described in the Materials and Methods section. (**A**) Real-time measurement of Shh substrate turnover kinetics of Hhat-enriched P100(sol) (45 µg/mL) at 1 µM Shh(1-10)-FAM and 3 µM palmitoyl-CoA. (**B**) Serial dilution of Hhat-enriched P100(sol) fraction performed as described for panel A. (**C**) Measurement of Shh substrate turnover of Hhat-enriched P100(sol) fraction (15 µg/mL) under stopped conditions by addition of nonfluorescent Shh(1-10)-K to 20 µM final concentration. (**D**) Stability of Shh(1-10)-FAM stopped signal from panel C as determined by real-time reaction monitoring for 1 h. Values are background corrected to heat-inactivated Hhat-enriched P100(sol) and represent mean ± SEM (assays performed in duplicate, *n* = 3).

The ability of Hhat to palmitoylate Shh(1-10)-FAM was tested using the voltages and pressure previously identified. For enzyme assays, the concentration of the Shh(1-10)-FAM peptide was quantified via FAM absorbance at 495 nm.^17^ Solubilized membrane fractions containing Hhat were prepared using DDM detergent according to literature protocols^11,13^ and subsequently enriched in Hhat through nickel affinity purification at 4 °C (**Suppl. Fig. S4**). Enzyme assays were performed by incubating Hhat-enriched P100(sol) fractions (45 µg/mL) with Shh(1-10)-FAM (1 µM) and initiated by addition of palmitoyl-CoA (3 µM). The reaction was monitored by sampling at 5 min intervals for 1 h, thus allowing real-time analysis of Shh palmitoylation. An increase in the palmitoylated Shh(1-10)-FAM (Pal-Shh(1-10)-FAM) product peak area with time, along with a corresponding decrease in the Shh(1-10)-FAM substrate peak area, indicated substrate turnover by Hhat. Peak area quantification demonstrated that the Hhat-catalyzed reaction proceeded at a linear rate for approximately 15 min (**Fig. 2A**). Serial dilution of the enriched Hhat fraction indicated 15 µg/mL of Hhat-enriched P100(sol) (~250 nM Hhat; **Suppl. Fig. S4**) provided approximately 15% turnover in 25 min at a linear rate, allowing analysis under initial velocity conditions (**Fig. 2B**). Incubation of the Hhat fractions at room temperature for 1 h prior to initiation of the palmitoylation reaction resulted in reduced substrate turnover, and similarly, serial dilutions of Hhat showed lower conversion plateaus, indicating limited enzyme stability over time (data not shown).

Real-time measurement of peptide lipidation is a powerful tool for analyzing enzyme-catalyzed reactions; however, for certain applications, it is advantageous or essential to analyze the reaction at a single time point under stopped conditions (for example, when screening large sample numbers in parallel). To halt the lipidation reaction, a 20-fold excess of nonfluorescent substrate peptide H_2_N-**CGPGRGFGKR**K-CONH_2_ (Shh(1-10)-K) was added to outcompete lipidation of Shh(1-10)-FAM. Addition of Shh(1-10)-K to a final concentration of 20 µM at designated time points followed by MSA analysis indicated a linear reaction progression over approximately 30 min (**Fig. 2C**), in agreement with prior kinetic studies under nonstopped conditions (**Fig. 2B**). To verify arrest of the Hhat-catalyzed reaction and assess the stability of the stopped signal, the stopped reaction was repeatedly sampled over 1 h. This demonstrated a good degree of signal stability over time, with an average signal drift of 0.04%/min and a non–statistically significant deviation from 0%/min in four out of five time points (**Fig. 2D**; **Suppl. Table S3**). Indeed, addition of excess nonfluorescent substrate may provide a generic method to achieve stopped-assay conditions for a range of modifications analyzed via the MSA format. The minor change in signal for Shh(1-10)-FAM may be attributed to *S*-oxidation of the terminal cysteine residue to the sulfonic acid, which possesses a similar retention time to the palmitoylated product (**Suppl. Figs. S1–S3**). Attempts to prevent Shh(1-10)-FAM oxidation through the use of DTT (1 mM), TCEP (1 mM), and degassed buffers were, however, unsuccessful.

### Determination of Kinetic Parameters and Dose-Response Analysis

**Figure 3.**
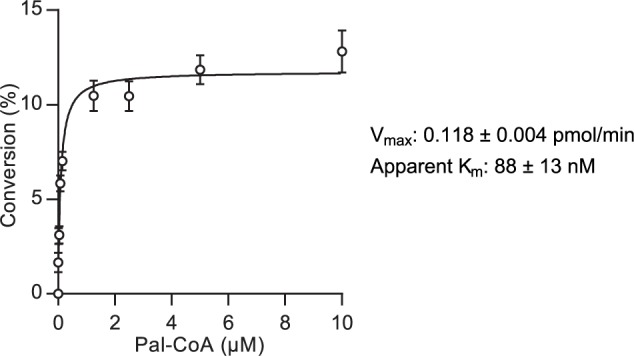
Kinetic analysis of Hhat measured by mobility shift assay. Kinetic analysis of Hhat and corresponding V_max_ and apparent K_m_ values. Assays were performed as described in **Figure 2C**, and the initial rate of Hhat-catalyzed Shh palmitoylation was measured as percentage conversion after 25 min in response to varied palmitoyl-CoA concentration with constant Shh(1-10)-FAM (1 µM) and Hhat-enriched P100(sol) fraction (15 µg/mL). An 11.8% conversion in 25 min (equivalent to 0.118 pmol/min) represents the V_max_ for the enzyme. Values represent mean ± SEM (assays performed in duplicate, *n* = 3).

Measurement of kinetic parameters in two substrate systems is typically performed by assessing the reaction rate in response to variation of one substrate concentration while the second substrate is held at a saturating concentration. The MSA format did not, however, allow for high concentrations of the fluorescently-labeled peptide owing to saturation of the detector system. The apparent K_m_ for palmitoyl-CoA at 1 µM Shh(1-10)-FAM was determined by measurement of the reaction rate over 25 min under stopped-assay conditions in response to variable palmitoyl-CoA concentration using 15 µg/mL enriched Hhat (**Fig. 3**). A V_max_ of 0.118 ± 0.004 pmol/min and apparent K_m_ of 88 ± 13 nM was recorded.

**Figure 4.**
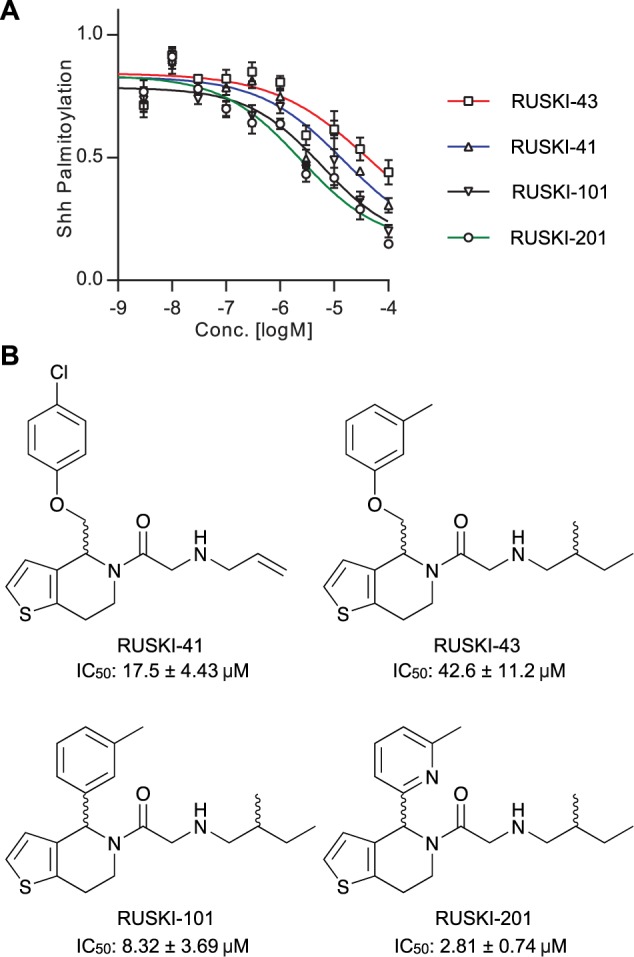
Dose-response analysis of Hhat inhibitors measured by mobility shift assay. (**A**) Dose-response curves for titration of RUSKI small-molecule inhibitors of Hhat. Assays were performed as described in **Figure 2C**, and inhibitors were tested over a seven-point, half-log unit serial dilution from 100 µM in a total DMSO volume of 250 nL. Assays were performed under stopped conditions, and samples were background corrected against heat-inactivated Hhat low control and normalized to DMSO vehicle-treated Hhat high control. RUSKI-201 showed the highest potency and RUSKI-43 the lowest potency of all inhibitors tested. (**B**) Structures of RUSKI inhibitors and corresponding IC_50_ values as determined by MSA.

The 5-acyl-6,7-dihydrothieno[3,2,*c*]pyridine (termed *RUSKI*) class of small-molecule inhibitors of Hhat were identified via a scintillation proximity assay high-throughput screen.^8^ We have previously reported the synthesis of four RUSKI inhibitors and measured their inhibitory activity against recombinant Hhat in our click-ELISA assay.^11,18,19^ Here, RUSKI-41, RUSKI-43, RUSKI-101, and RUSKI-201 were prepared as seven-point, half-log unit serial dilutions from 100 µM, and dose-response was measured via MSA under stopped-assay conditions. Response was corrected for background signal from reactions with heat-inactivated Hhat-enriched P100(sol) and normalized to DMSO vehicle-only control (**Fig. 4A**). All inhibitors exhibited IC_50_ values in the low µM range (**Fig. 4B**), and in agreement with previous analyses of these inhibitors, RUSKI-201 displayed the highest potency, followed by RUSKI-101 and RUSKI-41.^11^

## Discussion

In the current article, we present a method for assessment of peptide *N*-palmitoylation. The microfluidic MSA format exploited the differences in charge and hydrodynamic radius between the amine-substrate and amide-product peptides to facilitate quantification and was successfully applied to analyze palmitoylation of an N-terminal peptide from the protein Shh by the enzyme Hhat. This assay could also be applied to other forms of N-terminal acylation and has also recently been applied to analysis of lysine modification with small ubiquitin-like modifier or acetyl moieties.^23,24^ Although software is not currently available to predict optimal substrate sequences and screening conditions for fluorescent peak resolution for peptide *N*-lipidation, testing of differently charged peptide sequences under various voltages and pressures allows iterative identification of appropriate assay conditions. Development of such predictive software would be of significant benefit for future studies.

Existing lipidation assay formats using radiolabeled or bio-orthogonally-tagged lipids share the common limitation that they enable analysis of lipidation only under stopped-assay conditions.^11,13,25^ This is a result of the heterogeneous nature of these assays, which necessitates multiple steps, in contrast to the homogeneous nature of the MSA format. Alternative fluorescence-based homogeneous assay formats have been reported that allow real-time lipidation reaction monitoring; however, these formats employ measurement of the CoA by-product of the lipidation reaction. CoA is detected through use of thiol-reactive reagents, such as the fluorogenic maleimide derivative 7-diethylamino-3-(4-maleimido-phenyl)-4-methylcoumarin,^26^ or by enzyme-coupled reactions, which link CoA production to the fluorogenic reduction of NAD^+^.^27^ As such, these CoA detection assays do not represent a direct measure of lipidation, which may therefore render them more susceptible to false readings, for example, from nonspecific environmental effects on fluorescence,^28^ the presence of thiols or thiol-reactive groups,^29^ or inhibition of the enzyme-coupled reaction.^30^ In MSA, direct detection of the fluorescently-labeled lipidated product, in combination with nL “sip” volumes and the ability to perform repeated automated sampling of the reaction mixture, circumvents these existing assay limitations. To the best of our knowledge, the presented MSA format represents the first assay allowing direct, real-time measurement of peptide palmitoylation. The use of 20-fold excess unlabeled substrate peptide to block palmitoylation of the FAM-labeled substrate provides an effective and generic stop condition that can be applied to investigation of other enzymes. Stopped-assay conditions expand the sample base that can be analyzed by MSA and therefore allow application of this format to MTS campaigns.

The MSA format was also successfully applied to the analysis of Hhat kinetics and inhibitor dose response (**Fig. 3**). The apparent K_m_ recorded is in good agreement with the apparent K_m_ for DDM-solubilized Hhat of 150 ± 50 nM as measured by click-ELISA.^11^ However, these apparent K_m_ constants are in contrast to the *n*-octyl β-*D*-glucopyranoside (OTG)–solubilized Hhat apparent K_m_ of 3.0 ± 0.28 µM as recorded using a scintillation proximity assay.^13^ This difference may result from either more effective solubilization of palmitoyl-CoA by DDM compared with OTG or maintenance of a more active Hhat conformation in DDM. Small-molecule inhibitors showed the same ordering of potency as reported in the literature (**Fig. 4**)11; however, differences in absolute IC_50_ values relative to previous reports may result from use of an Hhat-enriched, and therefore more active, enzyme sample in the MSA assay. RUSKI-43, the most widely employed inhibitor in cell-based studies, showed the lowest potency of all compounds tested. The reported effects of this compound outside the canonical Hh pathway in cells demonstrate the need for improved understanding of the structure-activity relationship of these molecules.^12^ The MSA format presented here is a valuable tool in this process, as it provides a robust and cost-effective analysis of Hhat inhibition to facilitate further inhibitor development.

In summary, we have developed a microfluidic MSA format for measurement of *N*-palmitoylation that offers multiple advantages compared with existing assays. The MSA format does not employ radiolabeled substrates or require multiple liquid-handling steps and allows direct, real-time, and automated measurement of 12 samples in parallel. Addition of nonfluorescent substrate peptide provides a generic means to achieve stopped-assay conditions for application to screening of larger sample numbers. Both kinetic and dose-response analysis can be rapidly performed through this method, thus expanding the applications of MSA as a powerful tool in the analysis of amine posttranslational modification.

## Supplementary Material

Supplementary material
