# Supplementary material for: Microfluidic Mobility Shift Assay for Real-Time Analysis of Peptide N-Palmitoylation
Source: SLAS Discov. 2017 Jan 31;22(4):418–24. doi: 10.1177/2472555216689529 (PMC5453399; doi:10.1177/2472555216689529)
Supplement: Supplementary material [file Lanyon-Hogg_Supplemental_Material.pdf]

Supplemental Material for:

Microfluidic Mobility Shift Assay for Real-time Analysis of Peptide *N*-Palmitoylation

Thomas Lanyon-Hogg PhD,[1] Neki V. Patel PhD,[1,2] Markus Ritzefeld PhD,[1] Katherine J. Boxall,[3] Rosemary Burke PhD,[3] Julian Blagg PhD,[3] Anthony I. Magee PhD[*4] and Edward W. Tate PhD[*1]

Affiliation

[1] Department of Chemistry, Imperial College London, London, SW7 2AZ, UK; Tel: +44 (0)20 7594 3752. E-mail: e.tate@imperial.ac.uk

[2] Current address: Spirogen, QMB Innovation Centre, 42 New Road, London, E1 2AX, UK

[3] Cancer Research UK Cancer Therapeutics Unit, The Institute of Cancer Research, London, SW7 3RP, UK

[4] Molecular Medicine Section, National Heart & Lung Institute, Imperial College London, London, SW7 2AZ, UK. Tel: +44 (0)20 7594 3135. E-mail: t.magee@imperial.ac.uk

[*] Corresponding authors

Table of Contents

1. Supplementary Figures 2
2. Supplementary Tables 6
3. Materials 9
4. Abbreviations 9
5. Peptide Synthesis 9
6. Peptide Characterisation 10
7. Protein Analysis 17
8. References 17

1. Supplementary Figures

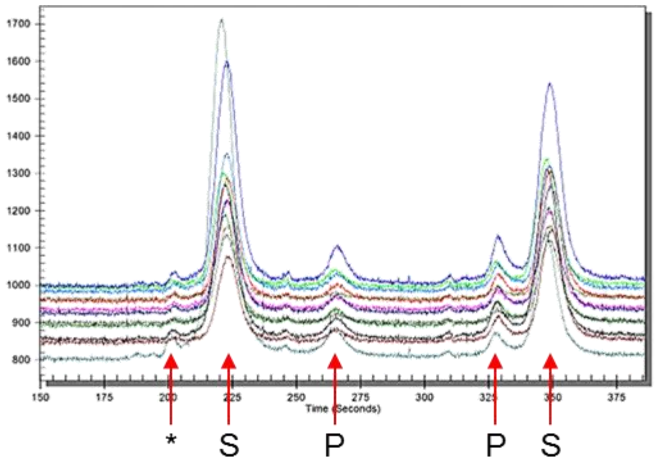

Figure S1. Fluorescence readings for separation of Shh(1-10)-FAM and Pal-Shh(1-10)-FAM. Three sample types were analysed; substrate Shh(1-10)-FAM (S), product Pal-Shh(1-10)-FAM (P), and substrate and product Shh(1-10)-FAM and Pal-Shh(1-10)-FAM mixture. Samples were 'sipped' at time points: S 180 s; P 240 s; S&P: 300 s. Samples were separated by MSA at -500 V downstream and -3000 V upstream voltage, at a screening pressure of -1.7 PSI. Fluorescence peak denoted * corresponds to *S*-oxidised sulfonic acid Shh peptide, resulting in a -1 charge difference thus corresponding to similar retention time to the *N*-palmitoylated product.

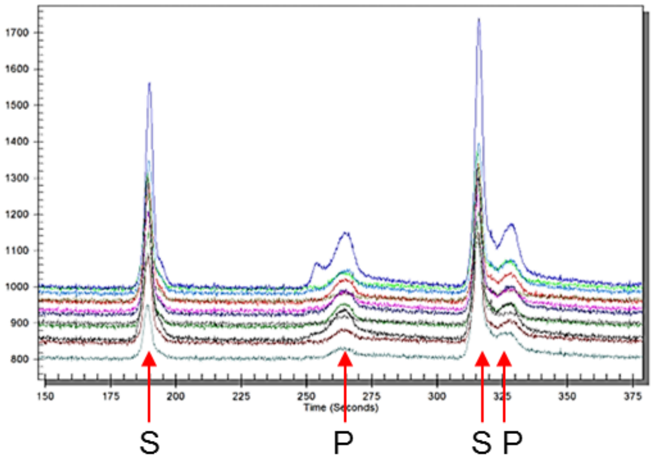

Figure S2. Fluorescence readings for separation of Shh(1-8AD)-FAM and Pal-Shh(1-8AD)-FAM. Sample mixtures and annotations are as described for Figure S1.

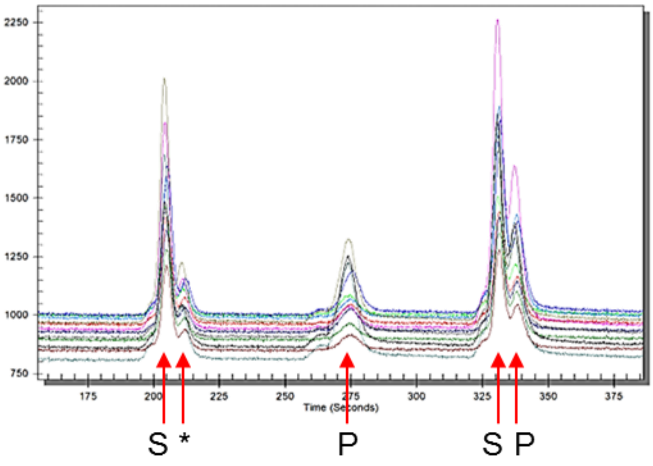

Figure S3. Fluorescence readings for separation of Shh(1-8)-FAM and Pal-Shh(1-8)-FAM. Sample mixtures and annotations are as described for Figure S1.

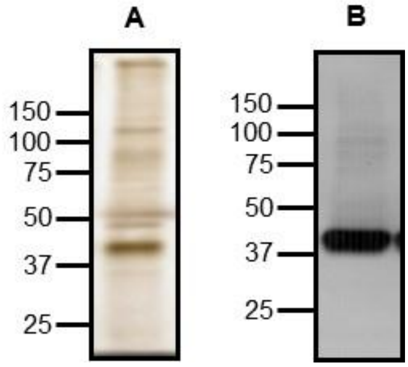

Figure S4. Hhat enrichment of P100(sol). P100(sol) fractions were prepared according to literature protocols[1,2] and enriched in Hhat-FLAG-His_8 *via* nickel affinity chromatography as described in Materials and Methods. (A) Silver stain indicating Hhat-enriched P100(sol) contains ~35% Hhat by densitometry. (B) α-FLAG-HRP immunoblotting.

2. Supplementary Tables

Peptide	Sequence	Substrate Charge	Product Charge
Shh(1-10)-FAM	H_2N-CGPGRGFGKR(K-FAM)G-$CONH_2$	+2	+1
Shh(1-8AD)-FAM	H_2N-CGPGRGFGAD(K-FAM)G-$CONH_2$	-1	-2
Shh(1-8)-FAM	H_2N-CGPGRGFG(K-FAM)G-$CONH_2$	0	-1

Table S1. Shh peptide sequences tested to identify appropriate MSA conditions. Overall charges are calculated at pH 7.4, assuming a FAM charge of -2 at this pH.[3]

Peptide	Downstream Voltage (V)	Upstream Voltage (V)	Screen Pressure (PSI)	Substrate Retention Time (s)	Product Retention Time (s)
Shh(1-8)-FAM	-500	-2250	-1.5	34	28
Shh(1-8)-FAM	-500	-3000	-1.7	30	35
Shh(1-8AD)-FAM	-500	-3000	-1.7	16	29
Shh(1-10)-FAM	-500	-3000	-1.7	48	26
Shh(1-10)-FAM	-500	-2000	-1.7	32	27
Shh(1-10)-FAM	-500	-1000	-1.7	23	19
Shh(1-10)-FAM	-500	-3000	-1.0	90	95
Shh(1-10)-FAM	-500	-3000	-2.5	8	3
Shh(1-10)-FAM	-500	-3000	-2.0	26	15
Shh(1-10)-FAM	-200	-3000	-1.7	48	27
Shh(1-10)-FAM	-200	-3000	-1.5	39	70

Table S2. Substrate-product peak separation under various screening pressures and voltages. As substrate peaks will be larger than product peaks in enzyme assays, elution of the product peak first aids in preventing overlap of peaks.

Time (min)	0	15	30	45	60
Gradient (%/min)	0.06 ± 0.02	0.04 ± 0.03	0.05 ± 0.03	0.04 ± 0.05	0.04 ± 0.03
Deviation from zero P value	0.0009	0.1207	0.1504	0.4905	0.1391
Deviation from zero significance	Significant	Not Significant	Not Significant	Not Significant	Not Significant

Table S3. Stopped signal stability analysis. Hhat-mediated Shh(1-10)-FAM palmitoylation was halted at indicated time points by addition of unlabeled Shh(1-10)K to 20 µM, and reaction mixtures then repeatedly sampled under continuous mode to determine stopped signal stability (Figure 3). A significant P value was taken to be < 0.05.

3. Materials

All reagents were purchased from commercial sources (Sigma-Aldrich, Fisher Scientific, Acros Organics, Novabiochem) and were used without further purification. High resolution mass spectrometry (HRMS) was performed using electrospray ionisation (ESI) on an AUTOSPEC P673 spectrometer.

4. Abbreviations

DCM (dichloromethane), DIPEA (*N*,*N*-diisopropylethylamine), DMF (dimethylformamide), DTT (dithiothreitol), FAM (5-carboxyfluorescein), HATU (1-[bis(dimethylamino)methylene]-1H-1,2,3-triazolo[4,5-b]pyridinium 3-oxid hexafluorophosphate), HBTU (*N*,*N*,*N′*,*N′*-tetramethyl-O-(1H-benzotriazol-1-yl)uronium hexafluorophosphate), ivDde (1-(4,4-dimethyl-2,6-dioxocyclohexylidene)-3-methylbutyl), NMM (*N*-methylmorpholine), NMP (*N*-methyl-2-pyrrolidone), PBS (phosphate buffered saline), PBS-T (phosphate buffered saline plus Tween-20 (0.1%)), TFA (trifluoroacetic acid), TIPS (triisopropylsilane).

5. Peptide Synthesis

All peptides were synthesised using an Intavis ResPep SL Automated Peptide Synthesiser (Intavis Bioanalytical Instruments, Germany) equipped with a Mini-Column array module, utilising the Fmoc/t-Bu solid phase orthogonal protocol. Resins (20 µmol) were swelled in DMF for 30 min prior to coupling. Fmoc-amino acid derivatives (100 µmol, 5 eq) pre-activated with HBTU (100 µmol, 5 eq) and NMM (200 µmol, 10 eq) in NMP were added, and incubated for 30 minutes at room temperature. The coupling mixture was removed by vacuum filtration before the coupling reaction was repeated. The peptide was capped with a 5% v/v acetic anhydride in NMP. The resin was washed with NMP and the Fmoc group removed by addition of piperidine in NMP (20% v/v). The resin was washed with DCM and DMF prior to the next coupling cycle. Substrate peptides were synthesised using *N*-α-Boc-protected terminal cysteine. Product peptides were synthesised using *N*-α-Boc-protected terminal cysteine, followed by Fmoc deprotection and coupling of palmitic acid using HATU activation and previously described coupling conditions. Fluorophore attachment was performed after the final peptide coupling *via* selective deprotection of *N*-ε-ivDde-protected lysine with hydrazine in DMF (5% v/v). FAM was activated using HATU and coupled using previously described coupling conditions. After completion of synthesis, the resin was washed with DMF, followed by DCM, followed by methanol, followed by diethyl ether and dried overnight under vacuum in a desiccator. Peptides were cleaved from resins in cleavage cocktail (1 mL, (v/v) TFA 83%, phenol 5%, H_2O 5%, 1 M DTT 5%, TIPS 2%) for 2 h at room temperature with agitation in the dark and the resin washed with cleavage cocktail (1 mL). The crude peptide was concentrated under a stream of N_2 for 15 min, and precipitated by dropwise addition to cold diethyl ether (-20 °C, 10 mL). Peptides were collected by centrifugation (4,000 rpm, 5 min, 4 °C), washed with cold diethyl ether (-20 °C, 4 x 10 mL) and dried in vacuo overnight. Peptides were analysed and purified using a Waters preparative RP-HPLC with a linear solvent gradient of 5-98% methanol (0.10% v/v formic acid) in water (0.10% v/v formic acid) over 18 min for synthetic substrate/product mixtures, or a linear solvent gradient of 20-98% methanol (0.10% v/v formic acid) in water (0.10% v/v formic acid) over 18 min for Shh(1-10)-FAM for Hhat enzyme reactions. The fractions containing purified peptide were combined and lyophilised.

6. Peptide Characterisation

Shh(1-10)K

LC-MS: RT = 0.8 min, MS (ESI, m/z, positive mode): calcd. for $C_{49}H_{85}N_{20}O_{11}S_1^{+}$: $[M+H]^+$ 1162.46, found: 1161.92 $[M+H]^+$, calcd. for $C_{49}H_{86}N_{20}O_{11}S_1^{2+}$ $[M+2H]^{2+}$ 581.73, found: 581.43, (ESI, m/z, negative mode) calcd. for $C_{49}H_{85}N_{20}O_{11}S_1^{-}$ $[M-H]^-$ 1160.44, found: 1159.90, calcd. for $C_{49}H_{84}N_{20}O_{11}S_1^{2}$: $[M-2H]^{2-}$579.72, found: 579.37.

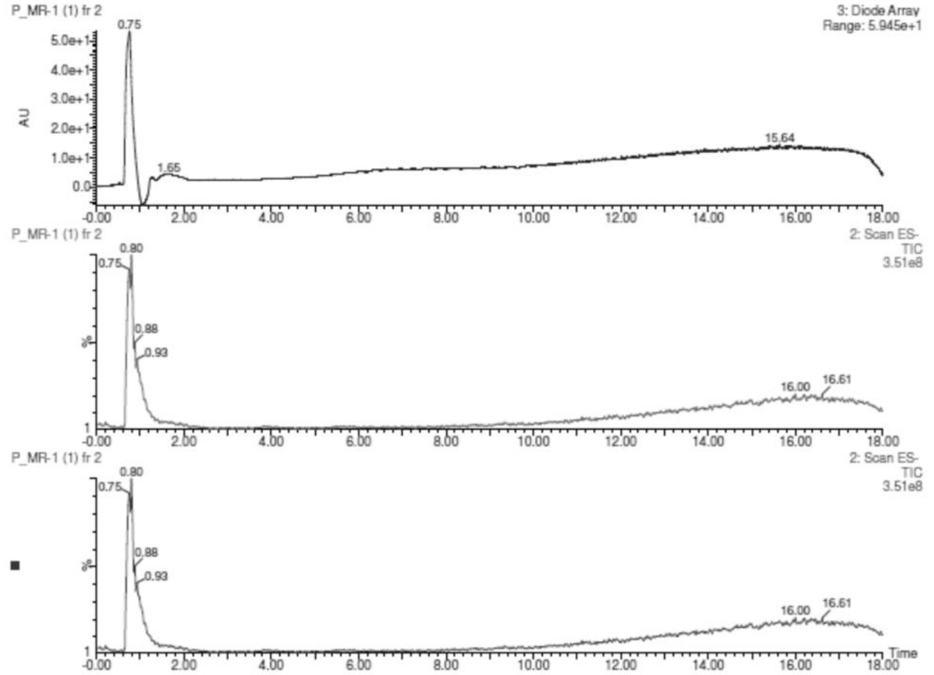

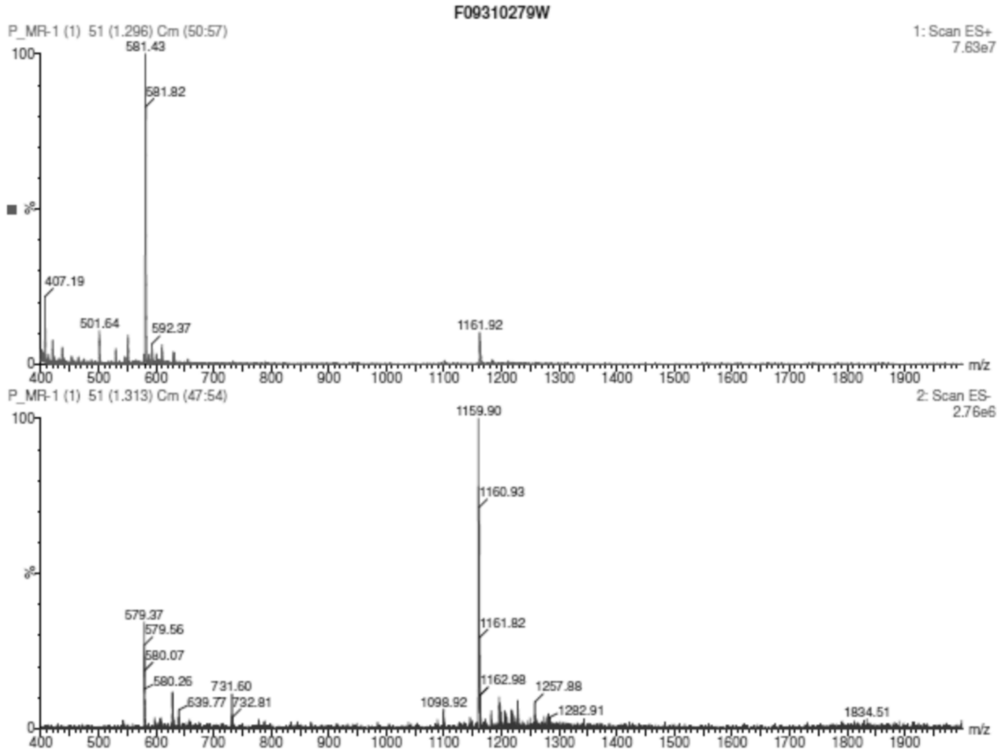

Shh(1-10)-FAM

LC-MS: RT = 8.00 min, MS (ESI, m/z, positive mode): calcd. for $C_{70}H_{96}N_{20}O_{17}S^{2+}$ $[M+2H]^{2+}$: 789.42, found: 788.82, calcd. for $C_{70}H_{99}N_{20}O_{17}S^{3+}$ $[M+3H]^{3+}$: 526.61, found: 526.28, (ESI, m/z, negative mode) calcd. for $C_{70}H_{95}N_{20}O_{17}S^{-}$ $[M-H]^{-}$: 1575,81, found: 1574,70, calcd. for $C_{70}H_{94}N_{20}O_{17}S^{2-}$ $[M-2H]^{2-}$: 787.40, found: 786.83.

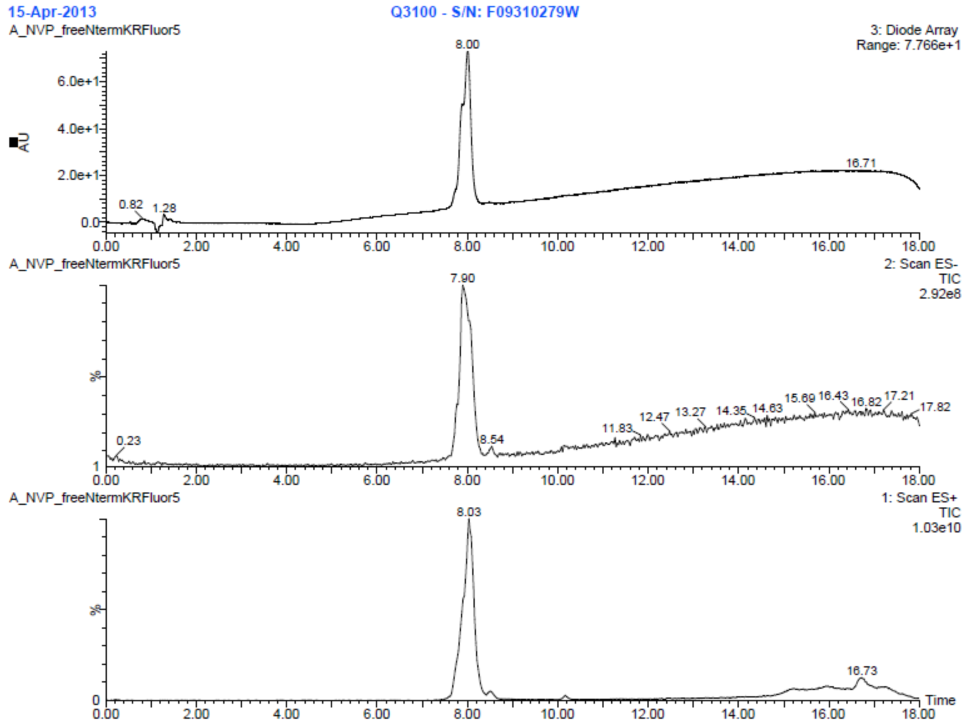

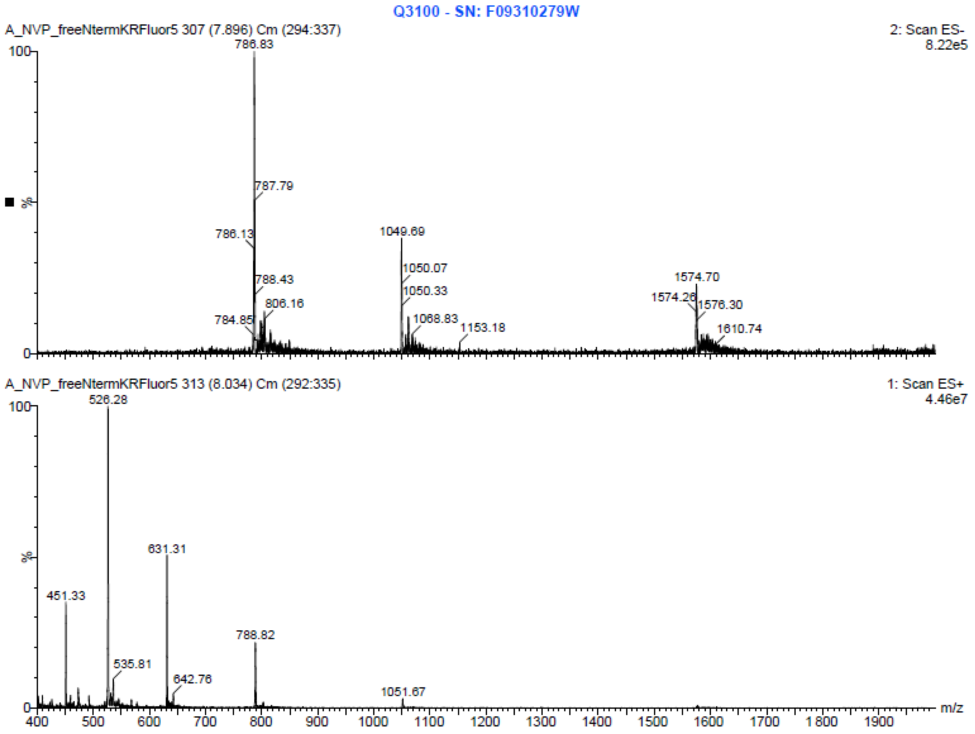

Pal-Shh(1-10)-FAM

LC-MS: RT = 13.04 min, MS (ESI, m/z, positive mode): calcd. for $C_{88}H_{129}N_{21}O_{19}S^{2+}$ $[M+2H]^{2+}$: 908.63, found: 907.99, calcd. for $C_{88}H_{130}N_{21}O_{19}S^{3+}$ $[M+3H]^{3+}$: 606.09, found: 605.70, (ESI, m/z, negative mode) calcd. for $C_{88}H_{126}N_{21}O_{19}S^{-}$ $[M-H]^{-}$: 1814.23, found: 1813.56, calcd. for $C_{88}H_{125}N_{21}O_{19}S^{2-}$ $[M-2H]^{2-}$: 906.62, found: 907.99.

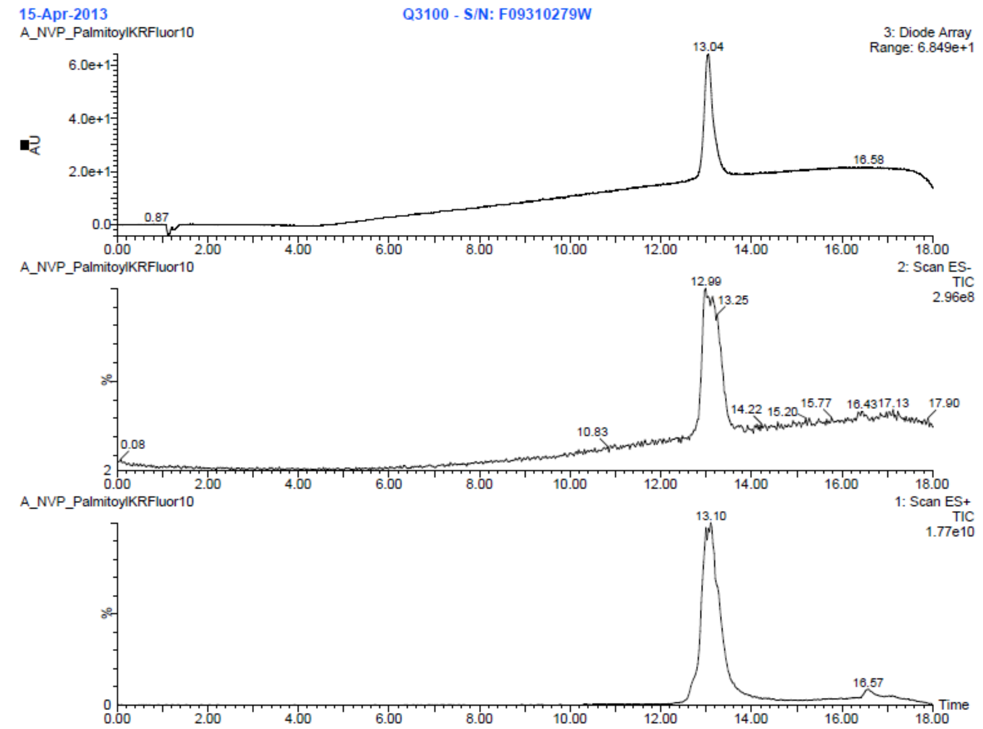

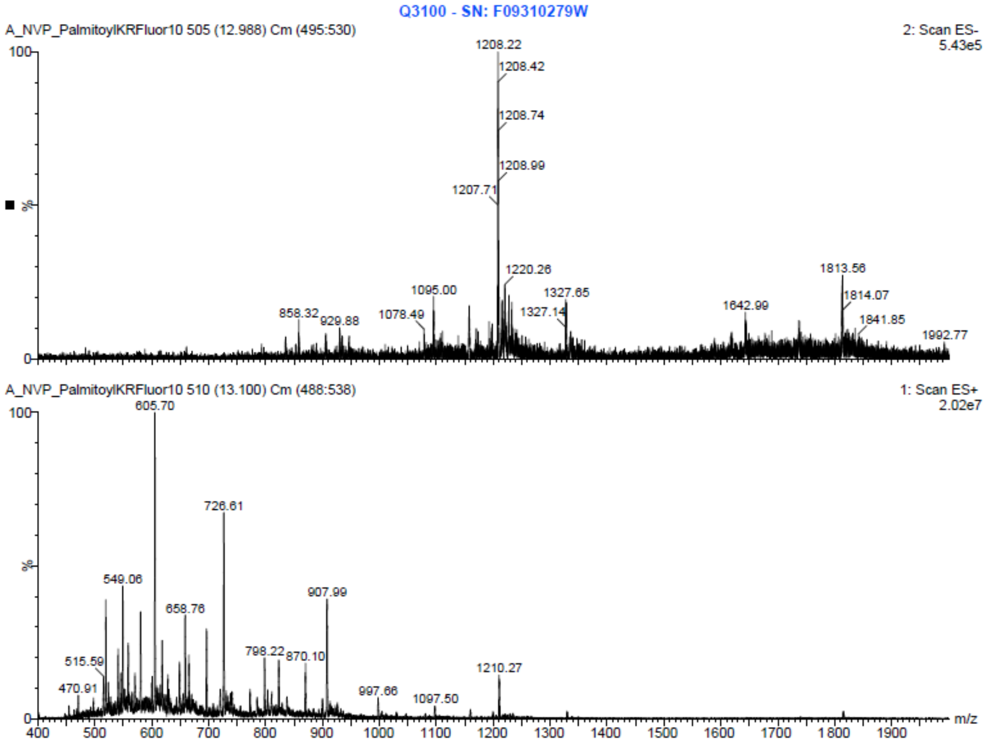

Shh(1-8AD)-FAM

LC-MS: RT = 10.18 min, MS (ESI, m/z, positive mode): calcd. for $C_{67}H_{84}N_{17}O_{20}S^{+}$ $[M+H]^{+}$: 1479.62, found: 1479.85, calcd. for $C_{67}H_{85}N_{17}O_{20}S^{2+}$ $[M+2H]^{2+}$: 740.32, found: 739.98, calcd. for $C_{67}H_{86}N_{17}O_{20}S^{3+}$ $[M+3H]^{3+}$: 493.88, found: 493.70 (ESI, m/z, negative mode) calcd. for $C_{67}H_{83}N_{17}O_{20}S^{-}$ $[M-H]^{-}$: 1477.61, found: 1476.65, calcd. for $C_{67}H_{82}N_{17}O_{20}S^{2-}$ $[M-2H]^{2-}$: 738.30, found: 737.93.

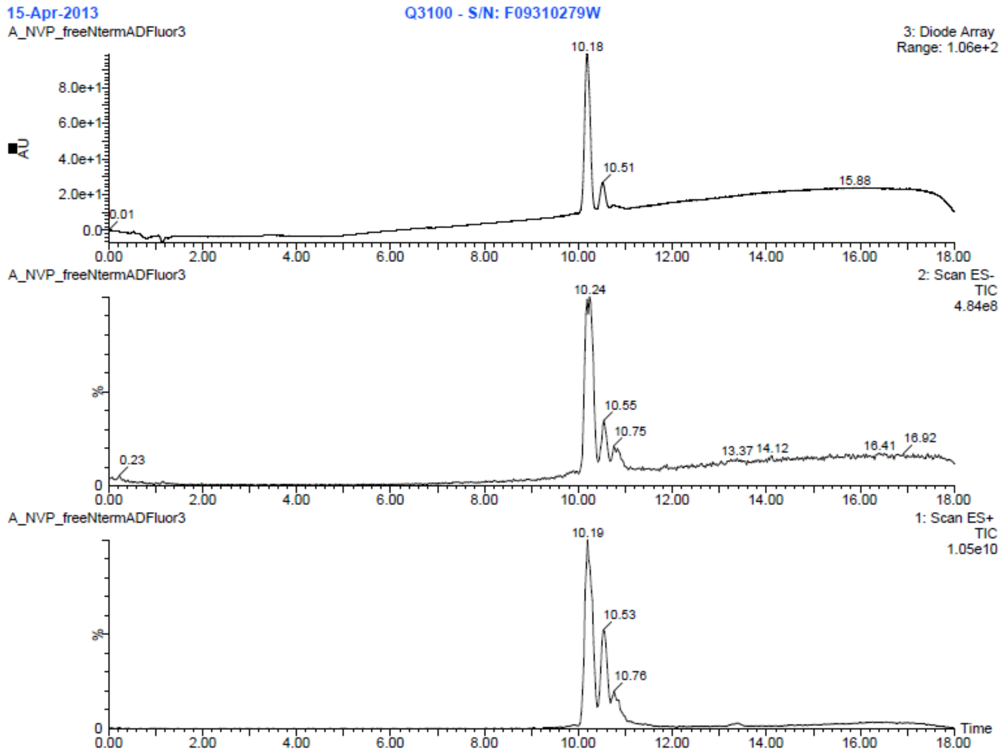

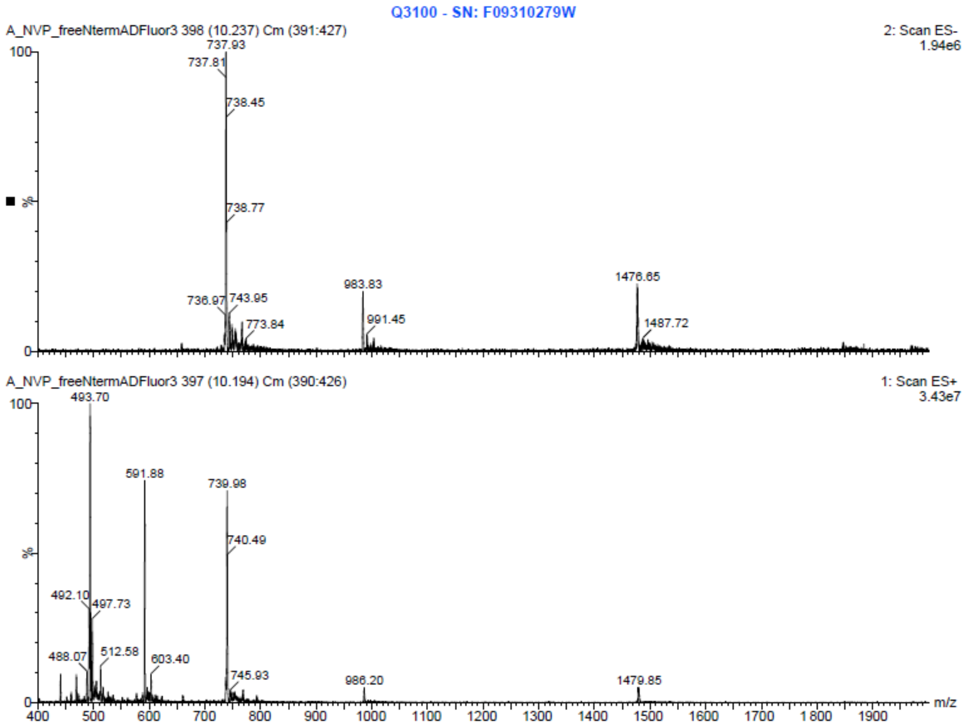

Pal-Shh(1-8AD)-FAM

LC-MS: RT = 14.66 min, MS (ESI, m/z, positive mode): calcd. for $C_{83}H_{113}N_{17}O_{21}S^{+}$ $[M+H]^{+}$: 1716.05, found: 1716.02, calcd. for $C_{67}H_{114}N_{17}O_{21}S^{2+}$ $[M+2H]^{2+}$: 859.53, found: 859.41 (ESI, m/z, negative mode) calcd. for $C_{67}H_{111}N_{17}O_{21}S^{-}$ $[M-H]^{-}$: 1716.03, found: 1716.02, calcd. for $C_{67}H_{110}N_{17}O_{21}S^{2-}$ $[M-2H]^{2-}$: 857.51, found: 857.17.

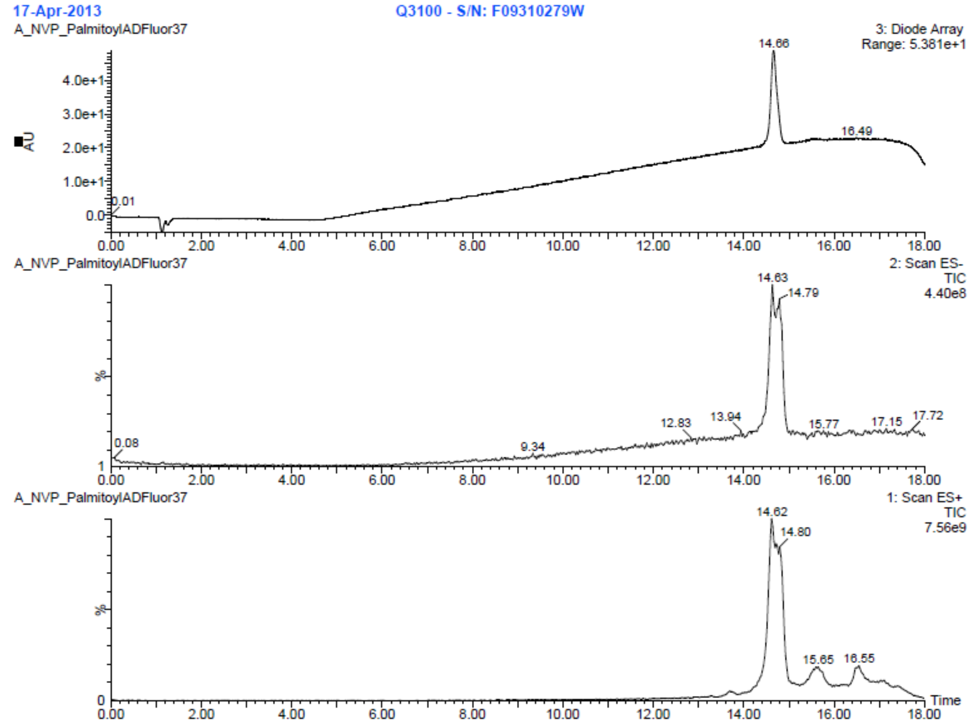

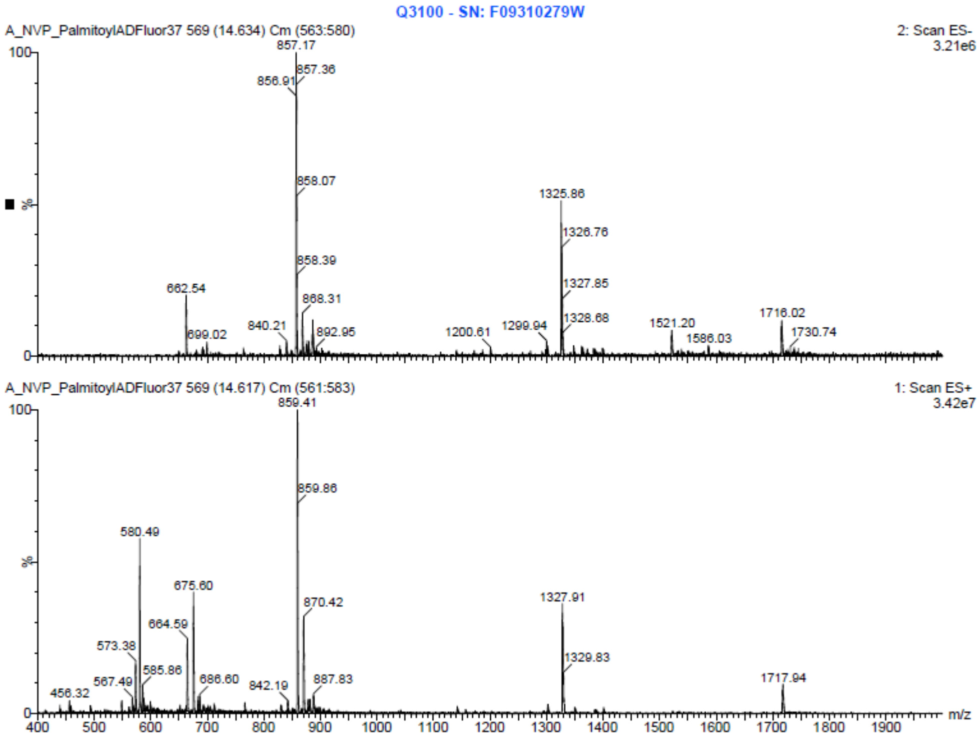

Shh(1-8)-FAM

LC-MS: RT = 10.31 min, MS (ESI, m/z, positive mode): calcd. for $C_{60}H_{74}N_{15}O_{16}S^{+}$ $[M+H]^{+}$: 1293.46, found: 1292.77, calcd. for $C_{60}H_{75}N_{15}O_{16}S^{2+}$ $[M+2H]^{2+}$: 647.23, found: 646.99 (ESI, m/z, negative mode) calcd. for $C_{60}H_{72}N_{15}O_{16}S^{-}$ $[M-H]^{-}$: 1291.44, found: 1290.66, calcd. for $C_{60}H_{73}N_{15}O_{16}S^{2-}$ $[M-2H]^{2-}$: 645.22, found: 644.87.

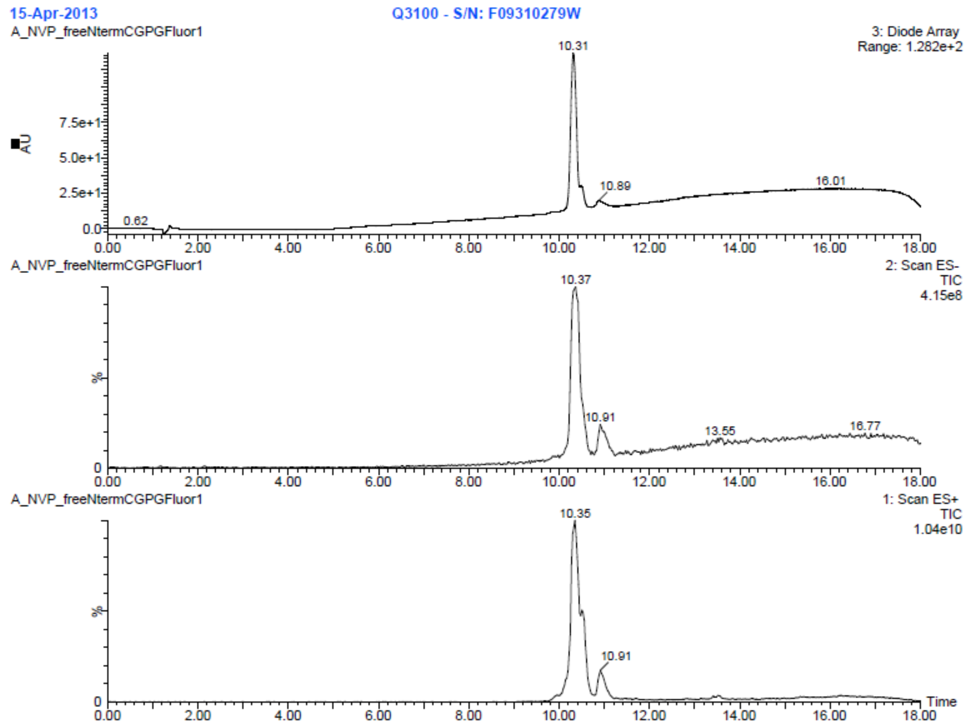

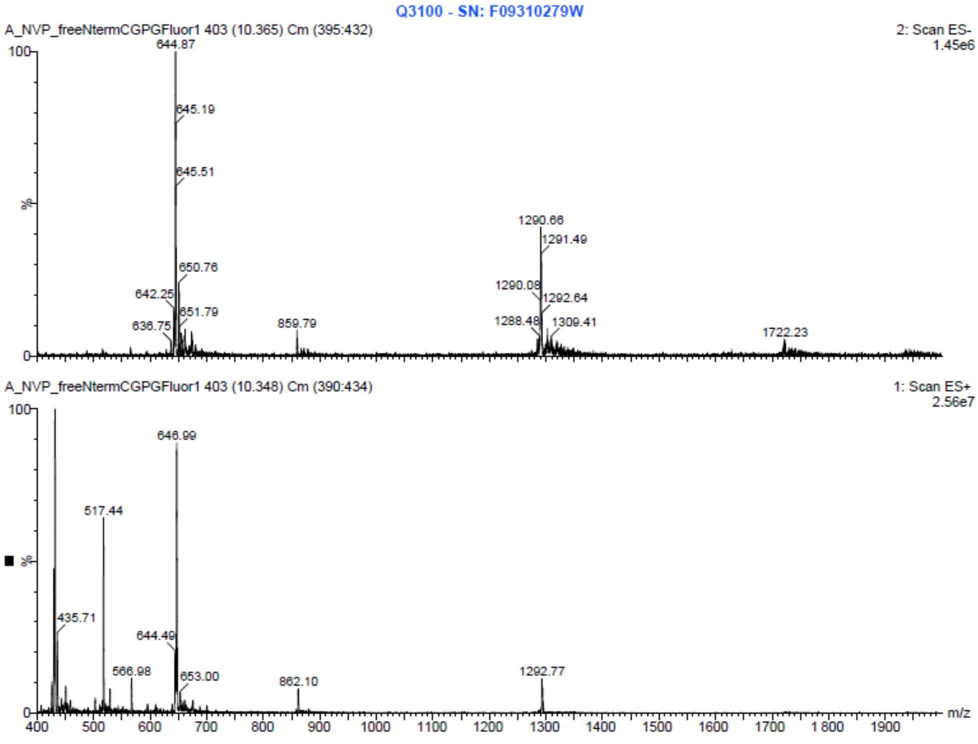

Pal-Shh(1-8)-FAM

LC-MS: RT = 14.65 min, MS (ESI, m/z, positive mode): calcd. for $C_{76}H_{104}N_{15}O_{17}S^{+}$ $[M+H]^{+}$: 1531.88, found: 1531.05, calcd. for $C_{76}H_{105}N_{15}O_{17}S^{2+}$ $[M+2H]^{2+}$: 766.44, found: 766.09 (ESI, m/z, negative mode) calcd. for $C_{76}H_{102}N_{15}O_{17}S^{-}$ $[M-H]^{-}$: 1529.87, found: 1529.77, calcd. for $C_{76}H_{101}N_{15}O_{17}S^{2-}$ $[M-2H]^{2-}$: 764.43, found: 764.05.

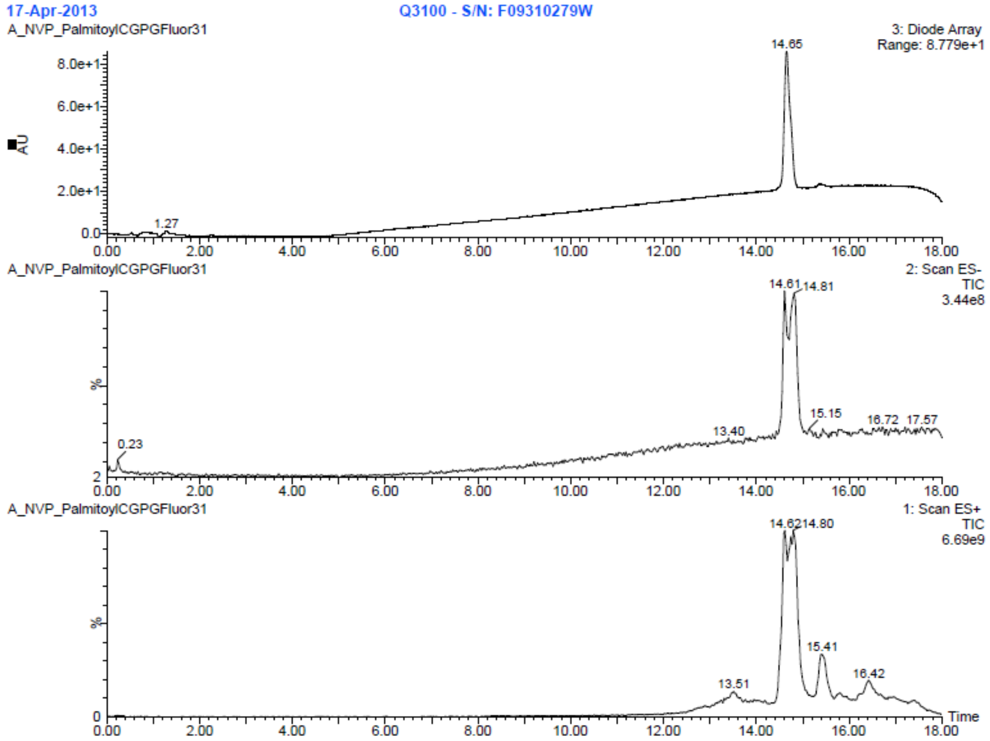

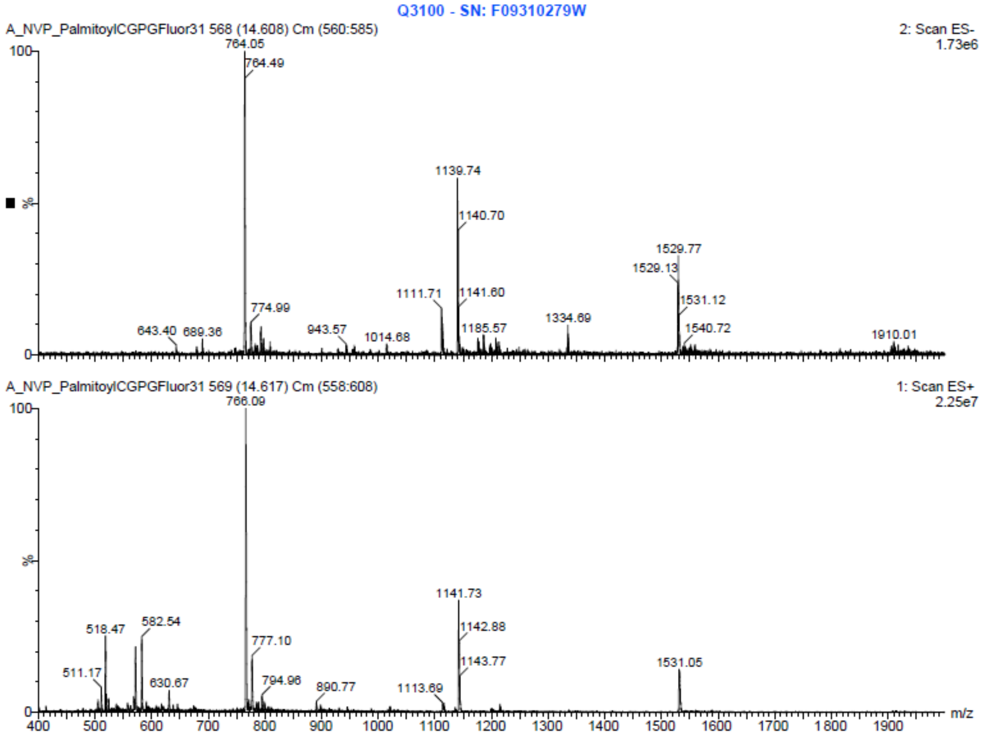

7. Protein Analysis

Protein samples were supplemented with reducing NuPAGE Sample Buffer (Fisher), separated by SDS-PAGE on a 15% gel without boiling the sample prior to analysis. Silver staining was performed using ProteoSilver™ Silver Stain Kit (Sigma-Aldrich) according to the manufacturer's guidelines. Gels were visualised on an Ettan DIGE Imager (GE Healthcare), and band intensity quantified by densitometry using ImageJ.

For immunoblot analysis proteins were transferred to PVDF membranes (Millipore). Membranes were blocked at room temperature for 1 h in PBS with 5% skimmed milk, and then incubated with α-FLAG-HRP monoclonal antibody (1:3,000) for 16 h at 4 °C. Blots were washed with 3 × PBS-T and bound immunocomplexes detected with ECL Plus (Pierce) and visualised as before.

8. References

1. J. A. Buglino and M. D. Resh, *J. Biol. Chem.*, 2008, **283**, 22076–22088.
2. T. Lanyon-Hogg, N. Masumoto, G. Bodakh, A. D. Konitsiotis, E. Thinon, U. R. Rodgers, R. J. Owens, A. I. Magee and E. W. Tate, *Anal. Biochem.*, 2015, **490**, 66–72.
3. R. Sjöback, J. Nygren and M. Kubista, *Spectrochim. Acta. A. Mol. Biomol. Spectrosc.*, 1995, **51**, L7–L21.
